# Transformation zone types: a call for review of the IFCPC terminology to embrace practice in low-resource settings

**DOI:** 10.3332/ecancer.2023.1612

**Published:** 2023-10-09

**Authors:** Kofi Effah, Ethel Tekpor, Comfort Mawusi Wormenor, Nana Owusu Mensah Essel

**Affiliations:** 1 Cervical Cancer Prevention and Training Centre (CCPTC), Catholic Hospital, Battor, PO Box 2, Battor, via Sogakope, Volta Region, Ghana; 2 Department of Emergency Medicine, College of Health Sciences, Faculty of Medicine and Dentistry, University of Alberta, 730 University Terrace, Edmonton, AB T6G 2T4, Canada; a https://orcid.org/0000-0003-1216-2296; b https://orcid.org/0000-0001-5494-5411

**Keywords:** transformation zone, uterine cervical neoplasm, early detection of cancer, colposcopy, visual inspection with acetic acid

## Abstract

Most cervical cancers develop in the transformation zone (TZ). Type 3 TZs, where the full circumference of the squamocolumnar junction (SCJ) is not visible pose problems during cervical screening with visual inspection methods, as (pre)cancerous lesions may be missed. Several practical strategies can be implemented to convert type 3 TZs into TZ 1 or TZ 2, including the use of an endocervical speculum or hygroscopic cervical dilators, opening the vaginal speculum more widely, skillful use of cotton-tipped applicators, performing colposcopy in midcycle, and use of oral or vaginal misoprostol and estrogen to ‘ripen’ the cervix. With the 2011 International Federation for Cervical Pathology and Colposcopy (IFCPC) terminology, settings with better resources to manipulate the cervix for a better view of the endocervical canal may assign patients to different categories from those in low-resource settings during a colposcopic examination. Here, we propose a colposcopic revision to the current IFCPC classification by segregating TZ 2 according to the extent of endocervical involvement and TZ 3 according to whether any attempt is made to open the endocervical canal, if such attempt(s) were successful, and the extent to which the practitioner can visualise parts of the uterine cervix beyond the border of the SCJ in the endocervical canal. In this proposed reclassification, TZ 2A has no part of the SCJ extending beyond 5 mm into the endocervical canal, whereas TZ 2B has part or all of the SCJ extending beyond 5 mm into the endocervical canal. TZ 3 is further subclassified into TZ 3A if the practitioner does not attempt to open the endocervical canal or the endocervical canal is opened, but not beyond 5 mm and TZ 3B if the full circumference cannot be visualised after opening the endocervical canal beyond 5 mm. We believe this revision will improve and better standardise the classification of TZ types, with huge implications for practice in low-resource settings, due to limited options for referral and treatment, to reduce the risk of missed cervical cancers and suboptimal treatment resulting from ablating lesions that extend too far into the endocervical canal.

## Introduction

A large majority of high-grade lesions of the uterine cervix arise within the transformation zone (TZ) [1]; thus, visual inspection of the entire TZ is an essential component of cervical pre(cancer) screening. The TZ refers to the aspect of the cervix that lies between the original and new squamocolumnar junctions (SCJs). With increasing age (usually after 45 years), the TZ moves into the endocervical canal. To promote the use of uniform terminology and practice during colposcopy, the International Federation for Cervical Pathology and Colposcopy (IFCPC) described the following classification for cervical TZs in 2011 [2, 3]. In this classification (Table 1), a type 1 TZ (TZ 1) is entirely ectocervical and its full circumference can be visualised; a type 2 TZ (TZ 2) has part or all of its SCJ within the endocervical canal, but can still be visualised all round; and a type 3 TZ (TZ 3) has part or all of the SCJ within the endocervical canal and cannot be visualised all round [3]. Among these types, TZ 3 (which occurs in approximately 10%–20% of women who present to be screened [4, 5]) poses problems during cervical screening with visual inspection methods (such as visual inspection with acetic acid (VIA) and mobile colposcopy (enhanced VIA)), as (pre)cancerous lesions may be missed [6]. In high-resource settings, endocervical curettage can be performed for histopathology which may pick up non-visible endocervical intraepithelial neoplasia, particularly among women with TZ 3 who have cervical canal atrophy [7]; however, this approach is challenging for low-resource settings because pathologists are scarce and there is a lack of equipment to analyse specimen obtained from endocervical curettage [8]. To mitigate this, several practical strategies can be implemented to convert these TZ 3s into lower forms (TZ 1 or TZ 2).

**Figure 1.**
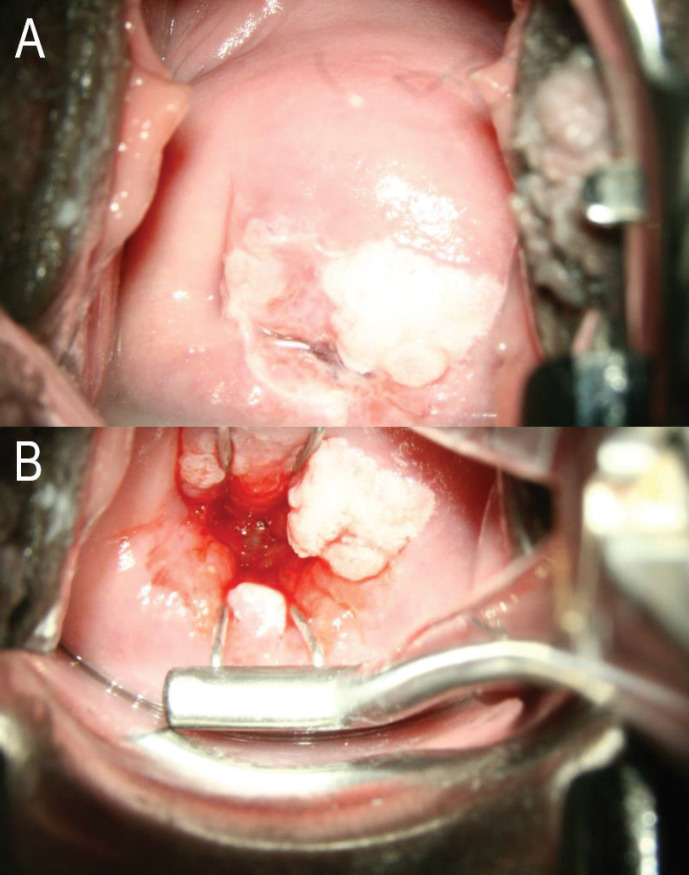
(a): No attempt made to open the endocervical canal. The entire circumference of the SCJ is not visible. This will be TZ 3A under the proposed reclassification. (b): Same woman. The entire circumference of the SCJ is visible using an endocervical speculum. The SCJ extends more than 5 mm into the endocervical canal. This will be TZ 2B under the proposed reclassification. Images courtesy CCPTC, Battor.

**Figure 2.**
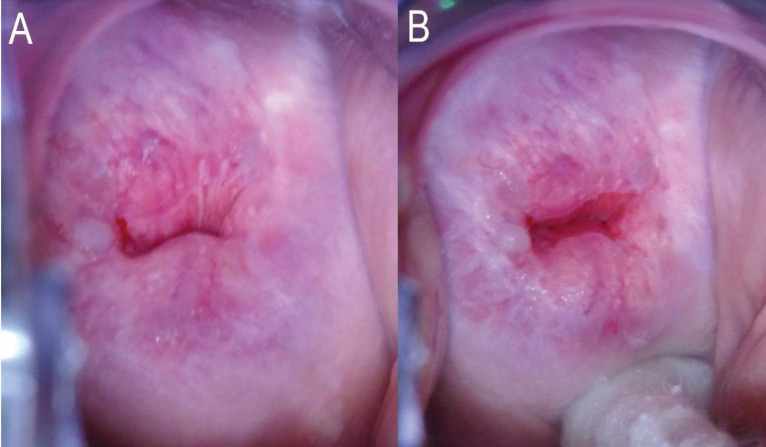
(a): No attempt made to open the endocervical canal. The entire circumference of the SCJ is not visible. This will be TZ 3A under the proposed reclassification. (b): Same woman. The entire circumference of the SCJ is visible after pushing a cotton swab into the posterior fornix of the vagina. The SCJ extends less than 5 mm into the endocervical canal. This will be TZ 2A under the proposed reclassification. Images courtesy CCPTC, Battor.

Such methods include the use of an endocervical speculum (Figure 1) or hygroscopic cervical dilators (such as Laminaria and Dilapan); opening the vaginal speculum more widely; skillful use of cotton-tipped applicators (Figure 2); performing colposcopy in midcycle when the endocervical canal is open and the mucus is clear; and use of oral or vaginal misoprostol and estrogen to ‘ripen’ the cervix [9–11]. Apart from opening the vaginal speculum widely and skillful use of cotton-tipped applicators, the other options may not be practical in low-resource settings especially in large population screening programs as it will be difficult to time women to be screened in midcycle. The use of endocervical specula will mean disinfecting the specula many times for mass screening which is not practical or using disposable specula which will add to costs. The nulliparous cervix may even not admit the speculum. The use of oral or vaginal misoprostol or hygroscopic dilators will require a repeat visit which may be difficult in settings that can only have visiting screening teams for a day. This may however be worth considering for community-based screening programs, like the Community-based Health Planning Services system in Ghana, where trained nurses or midwives are resident in the community, may have a community register, and have home visits as part of their schedules.

**Figure 3.**
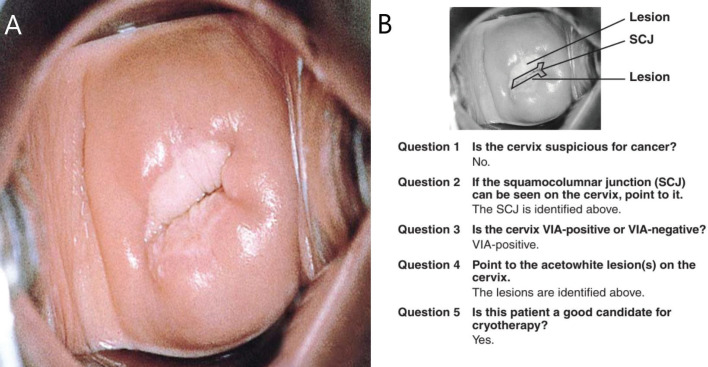
The JHPIEGO [16] visual inspection of the cervix flash card set ((A): front of flashcard 1 in the deck and (B): back of flashcard 1 in the deck) considers this woman a good candidate for cryotherapy. Under the proposed reclassification, this cervix would be TZ type 3A and would not qualify for ablative treatment. To be eligible for ablative treatment, an attempt should be made to see the full SCJ clearly in the endocervical canal (TZ 2A/2B) and the probe must completely cover the lesion/TZ during ablation. Source: Reproduced with permission from JHPIEGO Corporation.

Findings on VIA, which is a common screening method used in low-resource settings, are reported as negative, positive (for precancer), or suspicion for cancer [12]. It does not follow the IFCPC terminology in reporting TZ types. Since 2017, at our center (Cervical Cancer Prevention and Training Centre in Catholic Hospital, Battor, Ghana), our nurses and the over 320 health workers we have trained across Ghana and beyond, use the IFCPC terminology to describe the TZ types even when they perform VIA [13–15]. Also, mobile colposcopy (enhanced VIA) is increasingly being performed in low (middle) income countries by physicians as well as nurses and midwives [14] and the IFCPC terminology is used to report the findings thereof. We have found it easier and more practical to adopt the same terminology in teaching and reporting our findings in VIA and mobile colposcopy (enhanced VIA), as this ensures uniformity of practice. We believe this will be adopted globally in the near future. Further, the results of VIA and outcomes of ablative treatment (cryotherapy/thermal coagulation) have not been consistent globally; it depends on where one is trained. To illustrate this, the ‘JHPIEGO Visual Inspection of the Cervix Flash Card Set’, a leading resource used to train health professionals on VIA [16], recommends ablative treatment for the cervix shown in Figure 3. Our trained health workers would report this (at screening with either VIA or mobile colposcopy) as TZ type 3 (the full circumference of the SCJ not visible – upper limit of the lesion not seen); thus, this woman would not qualify for ablative treatment (cryotherapy or thermal coagulation). She has to be referred for excisional treatment like a loop electrosurgical excision procedure (LEEP).

## Problem statement

With the availability of the aforementioned methods of improving the visualisation of an otherwise partly- or fully-obscure TZ, the TZ type is no longer a non-modifiable screening characteristic. The goal of a terminology or classification is to create a consensus to guide management, standardise practice, and allow for comparison of outcomes across centers globally. The current IFCPC terminology does not do this as centers with better resources to improve TZ visualisation may assign patients to different categories from those in low-resource settings.

## Proposal for review of the IFCPC terminology

In light of the foregoing, we propose a colposcopic revision to the current IFCPC classification by segregating TZ 2 according to the extent of endocervical involvement and TZ 3 according to whether any attempt is made to open the endocervical canal, if such attempt(s) were successful, and the extent to which the practitioner can visualise parts of the uterine cervix beyond the border of the SCJ in the endocervical canal. In this proposed reclassification, TZ 2A has no part of the SCJ extending beyond 5 mm into the endocervical canal, whereas TZ 2B has a part or all of the SCJ extending beyond 5 mm into the endocervical canal. TZ 3 is further subclassified into TZ 3A if the practitioner does not attempt to open the endocervical canal or the endocervical canal is opened, but not beyond 5 mm and TZ 3B if the full circumference cannot be visualised after opening the endocervical canal beyond 5 mm (Table 2).

Women with fully visible TZ types (TZ 1 or TZ 2) on cervical examination can be successfully treated in a single visit (via thermal ablation or cryotherapy), whereas those with TZ 3 are generally considered ineligible for such procedures and should be referred for further examination (and may require, for example, LEEP to provide a histological diagnosis) [17]. This situation is especially important in low-resource settings in which options for referral are limited, further emphasising the need for reliable classification systems that allow TZ types to be accurately differentiated. With this reclassification, with resources (like an endocervical speculum) to probe the endocervical canal (Figure 1), TZ 3A could actually be TZ 2A or TZ 2B in a more resourced setting. Apart from the importance of this during screening and follow up of screen positives (visual inspection methods have limitations when the entire TZ is not visible), there are huge implications when it comes to treatment. High-grade lesions tend to extend along the crypts of the TZ and depths of up to 5 mm have been observed [18]; thus, any treatments should extend beyond a 5 mm depth. Where available, a marked probe (like a uterine sound) can be used to measure the 5 mm into the endocervical canal to assign the proposed (sub)types of TZ. Where a marked probe is unavailable, a subjective assessment may be made; 5 mm is about a quarter of a fingerbreadth.

The 2019 World Health Organization Guidelines for the Use of Thermal Ablation for Cervical Pre-Cancer Lesions [17] states that screen-positive women with no suspected invasive or glandular lesion (i.e., adenocarcinoma or adenocarcinoma *in situ*) are eligible to undergo thermal ablation if the TZ is entirely visible and does not extend into the endocervical canal; or the lesion is TZ 1; or the lesion is TZ 2, where the tip of the probe will yield complete ablation of the SCJ (reach the upper border of the TZ). Even if the TZ or SCJ extends into the endocervical canal, the TZ and lesion must be covered by the available probe in order to be eligible for ablation. Precancerous lesions extending more than 5 mm into the endocervical canal (the proposed types TZ 2B, 3B, and some 3A) may not receive optimal treatment with ablation (cryotherapy, thermal coagulation, laser vaporisation, or ablation with diathermy), especially if a flat (not ‘nipple’) probe is used for cryotherapy or thermal coagulation. Middle-cadre staff who treat precancerous cervical lesions in low-resource settings must understand these well and refer these women for excisional procedures like LEEP; otherwise, cases of treatment failure with persistent cervical intraepithelial neoplasia after ablative treatment would arise, possibly progressing to cancer. New automated visual inspection devices that use artificial intelligence hold promise and can be programmed to alert health workers about these to avoid treatment failures.

## Conclusion

Despite the availability of practical strategies to convert TZ 3 into TZ 2 or 1, the existing IFCPC terminology does not account for such approaches. Settings with better resources to improve TZ visualization may assign patients to different categories from those in low-resource settings. Here, we propose a colposcopic revision to the current IFCPC classification by segregating TZ 2 according to the extent of endocervical involvement and TZ 3 according to whether any attempt is made to open the endocervical canal, if such attempt(s) were successful, and the extent to which the practitioner can visualize parts of the uterine cervix beyond the border of the SCJ in the endocervical canal. We believe this revision will improve and better standardize the classification of TZ types, with huge implications for screening and treatment in low-resource settings, due to limited options for referral and LEEP, to reduce the risk of missed cervical cancers.

## Conflicts of interest

The authors declare that they have no conflict of interest.

## Funding

This work received no specific grant from any funding agency in the public, commercial, or not-for-profit sectors.

## Author contributions

Conceptualization: KE; Writing – original draft: NOME, KE, ET and CMW. All the authors read and approved the manuscript in its current form.

## Figures and Tables

**Table 1.** Current IFCPC nomenclature of TZ types.

TZ type	Definition
1	The entire circumference of the SCJ is visible, completely ectocervical.
2	The entire circumference of the SCJ is visible, partly or fully in the endocervical canal.
3	The entire circumference of the SCJ is not visible. The SCJ may be partly or fully in the endocervical canal.

TZ, transformation zone; SCJ, squamocolumnar junction

**Table 2.** Proposed reclassification/terminology of TZ types.

TZ type	Definition
1	The entire circumference of the SCJ is visible, completely ectocervical.
2	The entire circumference of the SCJ is visible, partly or fully in the endocervical canal.
2A	No part of the SCJ extends beyond 5 mm in the endocervical canal.
2B	Part of or all the SCJ extends beyond 5 mm into the endocervical canal.
3	The entire circumference of the SCJ is not visible. The SCJ may be partly or fully in the endocervical canal.
3A	No attempt made to open the endocervical canal (e.g., with an endocervical speculum) or endocervical canal opened but not beyond 5 mm.
3B	The entire circumference of the SCJ is not visible even after opening the endocervical canal beyond 5 mm.

TZ, transformation zone; SCJ, squamocolumnar junction
